# Development and Design of a Pediatric Case-Based Virtual Escape Room on Acute Iron Toxicity

**DOI:** 10.5070/M5.52192

**Published:** 2026-01-31

**Authors:** Kaitlyn Boggs, Manu Madhok, Tania Ahluwalia

**Affiliations:** 1 Medical University of South Carolina, Division of Pediatric Emergency Medicine, Charleston, SC; 2 Children’s Minnesota, Division of Emergency Medicine, Minneapolis, MN; 3 University of Washington/Seattle Children’s Hospital, Division of Emergency Medicine, Seattle, WA

## Abstract

**Audience:**

This virtual escape room (VER) serves as a didactic activity tailored for learners specializing in emergency medicine, pediatrics, and family medicine across all postgraduate years. The VER can be undertaken collaboratively in teams or individually, leveraging virtual platforms and adaptable to various educational settings.

**Introduction:**

Iron tablets appeal to children due to their vibrant color and sugar coating, resembling candy. Nearly 11,000 cases of iron exposure in children under six are reported annually in the US.^1^ More severe incidents involve prenatal vitamins and iron preparations containing ferrous sulfate, which has a significantly higher concentration of elemental iron per tablet than other formulations.^2^ Virtual escape rooms (VERs) are an innovative educational tool for teaching about acute iron toxicity. By integrating gamification into medical education, VERs offer a unique approach as participants can join remotely and interact with a team of other learners in geographically distinct locations.

**Educational Objectives:**

By the end of the activity, learners should be able to:

1. Recognize the history and clinical presentation of acute iron toxicity
2. Demonstrate knowledge of the necessary workup in suspected iron toxicity
3. Identify the stages of acute iron toxicity
4. Identify management of iron toxicity and its complications
5. Perform appropriate management in the setting of decompensated hemorrhagic shock and hypovolemia
6. Demonstrate teamwork through communication and collaboration

**Educational Methods:**

The development process encompassed a seven-step approach: creating a scenario, defining learning objectives, and designing a suitable room.^3^ Clues and puzzles aligned with the specified learning objectives. The VER was hosted on Articulate 360 (Articulate Global Inc.) and complemented by a facilitator guide that provided content and technical support.

**Research Methods:**

To replicate this activity, a team of facilitators should be present to organize the participants into small groups and distribute the VER link. During our implementation, this link was shared in real-time on Zoom Video Communications Inc. (Zoom), leveraging breakout rooms to assign participants to their respective rooms. Additionally, we conducted this in person with faculty and nursing, where participants were divided into groups accordingly. There was a structured format: pre-briefing, a timed escape room scenario, debriefing, and evaluation. Afterward, learners evaluated the VER and educational content with a survey hosted on Google Docs (Google LLC).

**Results:**

A total of 55 respondents completed post-evaluation surveys. Despite limited experience with previous virtual escape rooms, both trainees and faculty agreed the design was easy to follow (78.2%), fostered teamwork (90.9%), and was a feasible method of education (85.5%).

**Discussion:**

This activity was successfully implemented with trainees, faculty, and nursing professionals, demonstrating the ability of VER to be utilized in a wide variety of applications. We also successfully implemented this format in both in-person and online platforms. Limitations of this include a need for long-term outcome data. Future studies could further assess knowledge improvement and clinical management of acute iron toxicity.

**Topics:**

Acute iron toxicity, emergency medicine, escape room, ingestion, gamification, pediatrics, toxicology, virtual escape rooms.

## USER GUIDE

**Table:** 

List of Resources:	
Abstract	1
User Guide	3
Small Groups Learning Materials	6
Appendix A: Facilitator Guide	6
Appendix B: Key Clinical Pearls	23
Appendix C: Survey	27

**Learner Audience:**

This VER is directed toward interns, junior and senior residents, attendings, and simulation educators.

**Time Required for Implementation:**

These VER sessions were modeled similarly to telesimulations. Telesimulation is a methodology that uses internet-based communication technologies to deliver simulation-based education between instructors and learners in different locations, enabling real-time observation, interaction, and feedback.4 Each VER session included a pre-brief followed by a scenario, debrief, education, and evaluations. Each session lasted 90 minutes and was divided into specific segments: a 10-minute pre-brief introducing the VER and its platform, 60 minutes allocated for participants to engage in the escape room challenge, and a concluding 20-minute debrief session. Time was allotted to accommodate clues involving quantitative reasoning, which generally required more time for participants to solve than recognition-based questions. These challenges were designed to foster deeper discussions and teamwork among participants. Sixty minutes for the virtual escape room allows the time required for collaboration, problem-solving, and the use of the hint button when necessary.

**Recommended Number of Learners Per Instructor:**

All participants underwent randomization, facilitated either through Zoom’s breakout room feature or in-person allocation. One facilitator was assigned to each small group comprising 6–10 participants. Careful consideration was given to ensure representation across all post-graduate years or a mix of faculty within each group. Each group was assigned a dedicated facilitator to provide support and technical assistance throughout the session. Participants were encouraged to have their cameras turned on during the online session, adhering to a closed-book teaching format.

**Topics:**

Acute iron toxicity, emergency medicine, escape room, ingestion, gamification, pediatrics, toxicology, virtual escape rooms.

**Objectives:**

By the end of this activity, learners should be able to:

1. Recognize the history and clinical presentation of acute iron toxicity
2. Demonstrate knowledge of the necessary workup in suspected iron toxicity
3. Identify the stages of acute iron toxicity
4. Identify management of iron toxicity and its complications
5. Perform appropriate management in the setting of decompensated hemorrhagic shock and hypovolemia
6. Demonstrate teamwork through communication and collaboration

### Linked objectives and methods

The VER provides an innovative way to provide education on acute iron toxicity. This format was selected to make teaching more engaging for the learners. Our team established a sevenstep process, using a facilitator guide to streamline each stage.^3^ Initially, we identified acute iron toxicity as a high-yield topic and developed a case scenario, including patient presentation, physical examination, initial workup and evaluation, and acute management. Subsequently, we developed specific learning objectives to link to each of our clues. Third, we designed a contextually relevant room, drawing inspiration from real patient settings. We formatted the VER using Articulate 360, a subscription-based software for course creation, allowing for the addition of interactive activities, quizzes, and customization. Through our work with previous virtual escape rooms, we utilized feedback to transition to this platform with the assistance of our e-learning development experts.^5^ Fourth, we developed clues aligned with the learning objectives, incorporating interactive puzzles to enhance participant engagement. Including diverse puzzle choices, each containing a concealed key, facilitated participants’ progression through the sequence of clues. A hint document, including reference charts, was made available within the interface if participants were stuck on a clue for greater than two minutes. Fifth, our e-learning team embedded the clues into Articulate 360. Concurrently, the facilitator guide underwent refinement to include comprehensive content and technological support. Lastly, a pre-workshop meeting convened all facilitators to review content and technical aspects. Before implementation, the VER underwent trials with faculty members. This study was IRB-exempt.

### Recommended pre-reading for facilitator

Facilitators were required to have a comprehensive understanding of acute iron toxicity. All facilitators were trained in pediatric emergency medicine and interested in simulation-based education. Before the live session, each facilitator reviewed the facilitator guide, which included learning objectives, clue locations within VER, and solutions to the clues. Furthermore, facilitators underwent a trial of the VER to acquaint themselves with its layout and functionalities. Facilitators were requested to use a laptop or desktop computer for optimal performance during the session. We recommend 1–2 hours of preparation to review the facilitator guide, explore the VER platform, and familiarize themselves with the clues prior to leading a session.

### Small group application exercise (sGAE)

See the following attached materials for this small group exercise

- Appendix A: Facilitator Guide
- Appendix B: Key Clinical Pearls
- Appendix C: Survey

### Results and Tips for Successful Implementation

#### Pre-brief and Debrief

We recommend a pre-brief to orient all participants to the platform, minimize technological challenges, and discuss the activity’s goals. In addition, we recommend a standard debrief to discuss group collaboration, barriers noted throughout the activity, and key learning points. We distributed a survey following the completion of our activity to assess for additional feedback. The survey included a 5-point Likert scale and open-ended questions related to the satisfaction of the acute iron toxicity escape room (Appendix C).

#### Faculty Evaluations

Faculty participated in pilot sessions conducted for testing and feedback. This was trialed at the International Pediatric Simulation Symposia and Workshop (IPSS-W) conference in person with 27 survey respondents, including physicians (n = 20), nurses (n = 5), simulation educators (n = 1), and other allied health professionals (n = 1). All respondents (100%) agreed or strongly agreed that this model provided engaging education.^5^ Additionally, 96% expressed willingness to participate in a VER in the future, and 96% agreed or strongly agreed that they would integrate VER into their training sessions.^5^ Comments from respondents highlighted the advantages of the virtual format, emphasizing its easy accessibility and user-friendly interface. Many appreciated the opportunity to explore the platform and noted its role in fostering interactive learning, discussion, and teamwork. Others found it engaging and effective for imparting new knowledge. Team building and collaboration were also noted positively, enhancing the overall learning experience.

#### Trainee evaluations

**Graph 1:**
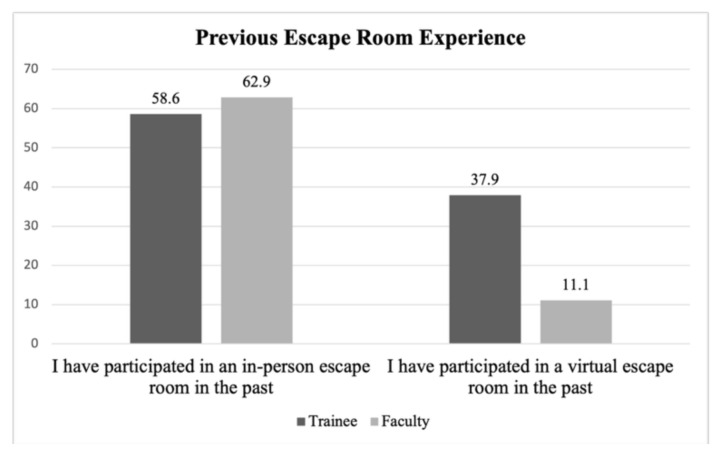
Previous Escape Room Experience

**Graph 2:**
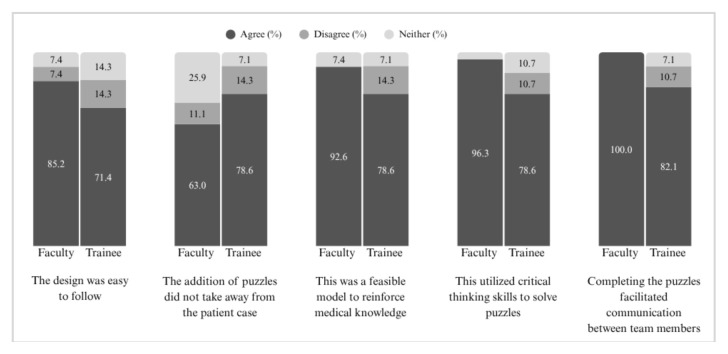
Evaluation of Acute Iron Toxicity Virtual Escape Room

This VER was also trialed with emergency medicine (EM) trainees with 29 survey respondents from all post-graduate levels (PGY-1: n = 10, PGY-2: n= 8, PGY-3: n = 11). Notably, this educational VER platform had been trialed with some trainees the previous academic year (Graph 1) and overall had more experience with VERs than the faculty group. In this VER, faculty acted as facilitators and were explicitly instructed to encourage discussion and teamwork rather than directly provide answers. Similar to the faculty evaluation, trainees appreciated the promotion of teamwork. Others highlighted that the platform was “novel,” “new,” and “game-like.” One commented that it “helped me gain knowledge in a fun way.” Many commented on the puzzles as an additional fun component to their education. Trainees also agreed they would participate in another VER in the future (79%) and that it identified gaps in knowledge of the topic presented (72%). Additional evaluation results can be found in Graph 2.

#### Discussion

Virtual escape rooms may be utilized for education gamification. Key findings included high satisfaction and engagement from both our faculty and trainees, with 100% of faculty and 79% of trainees expressing interest in future participation. The VER was praised for promoting collaboration, critical thinking, and a fun learning environment. Limitations include the small sample size and potential variability in prior experience with VERs. Future VERs should explore long-term knowledge retention to validate its educational impact.

## Figures and Tables

**Figure:**
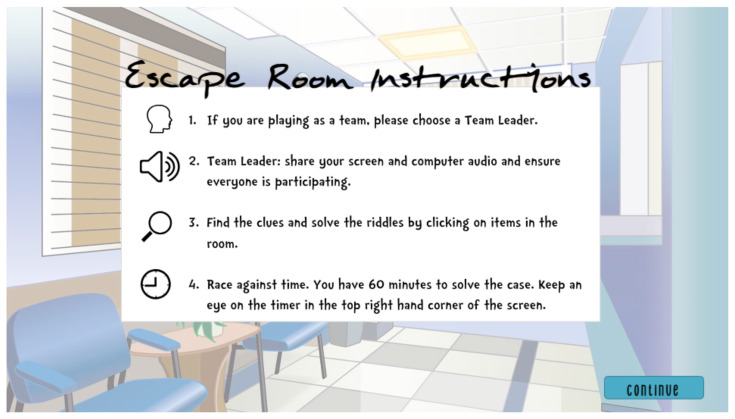
Children’s National E-Learning Team. Escape Room Instructions. In: LearnMedEd Escape Room #2, https://www.learnmeded.org/mod/scorm/view.php?id=624

**Figure:**
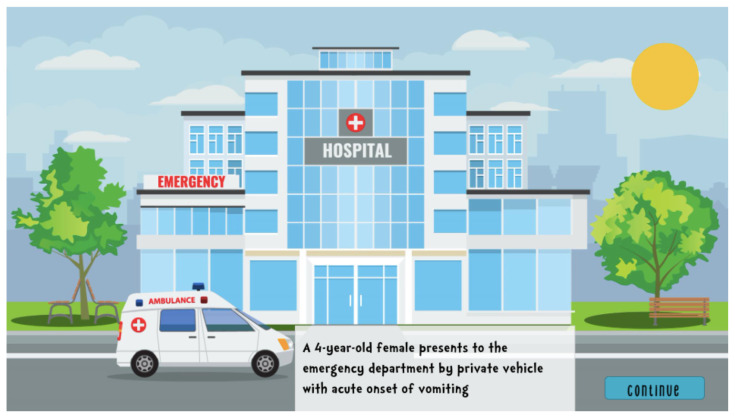
Children’s National E-Learning Team. Chief Complaint. In: LearnMedEd Escape Room #2, https://www.learnmeded.org/mod/scorm/view.php?id=624

**Figure:**
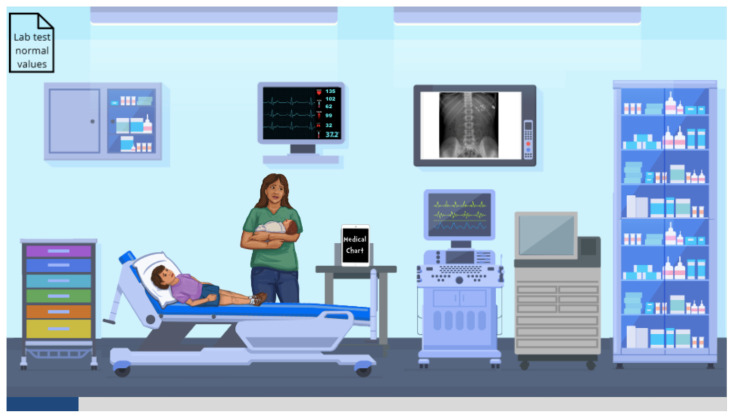
Children’s National E-Learning Team. Escape Room Home Screen. In: LearnMedEd Escape Room #2, https://www.learnmeded.org/mod/scorm/view.php?id=624

**Figure:**
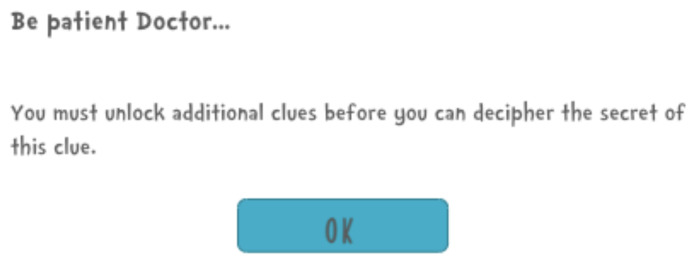
Children’s National E-Learning Team. Error Message. In: LearnMedEd Escape Room #2, https://www.learnmeded.org/mod/scorm/view.php?id=624

**Figure:**
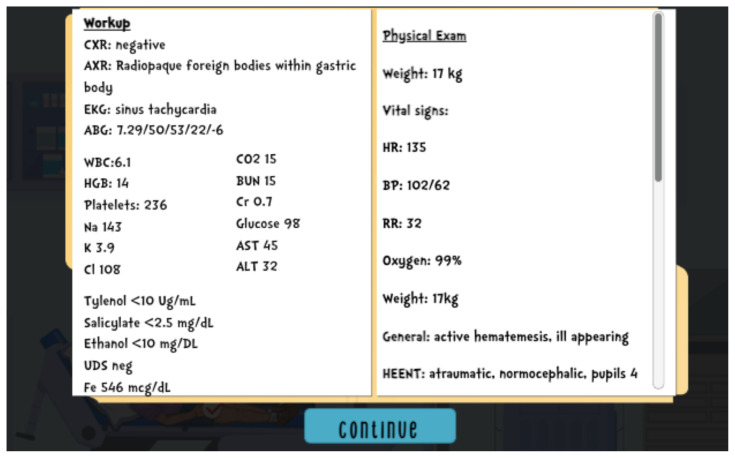
Children’s National E-Learning Team. Medical Chart. In: LearnMedEd Escape Room #2, https://www.learnmeded.org/mod/scorm/view.php?id=624

**Figure:**
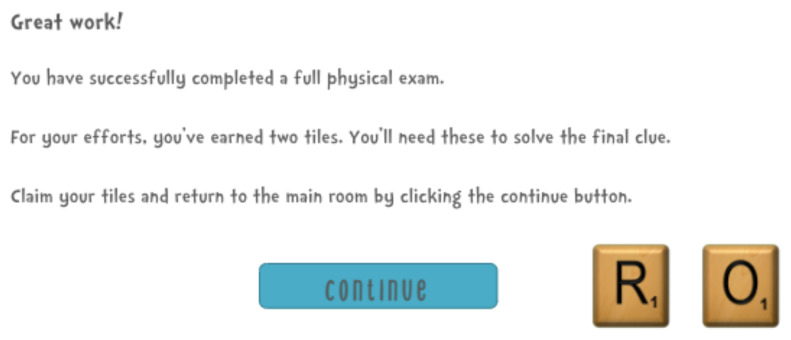
Children’s National E-Learning Team. Tile Collection. In: LearnMedEd Escape Room #2, https://www.learnmeded.org/mod/scorm/view.php?id=624

**Figure:**
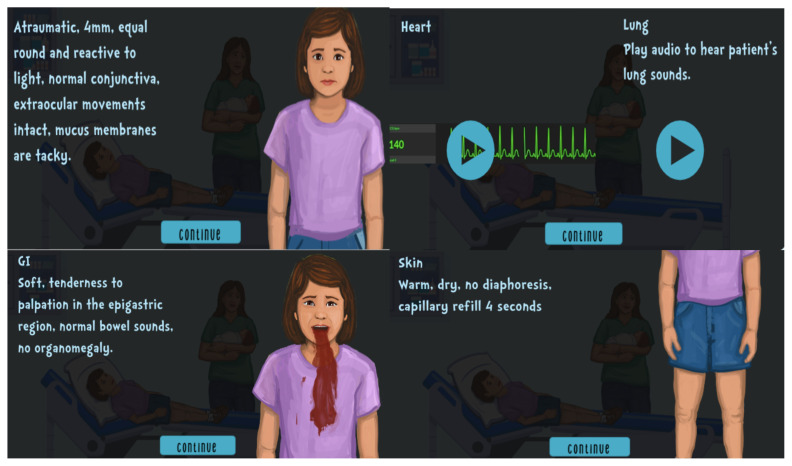
Children’s National E-Learning Team. Physical Exam. In: LearnMedEd Escape Room #2, https://www.learnmeded.org/mod/scorm/view.php?id=624

**Figure:**
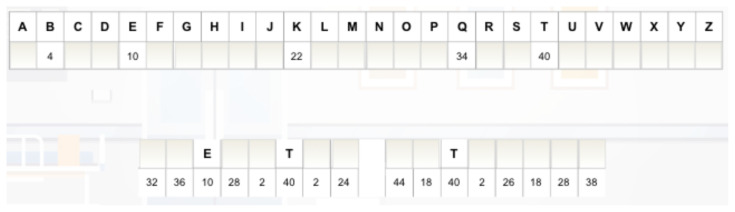
Children’s National E-Learning Team. Clue 2. In: LearnMedEd Escape Room #2, https://www.learnmeded.org/mod/scorm/view.php?id=624

**Figure:**
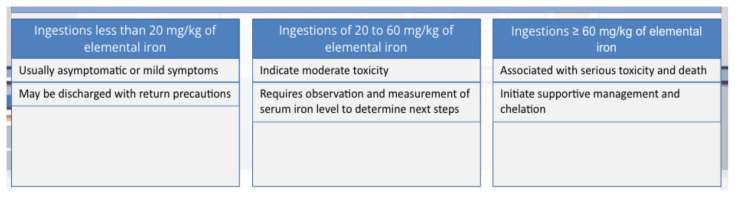
Children’s National E-Learning Team. Clue 4. In: LearnMedEd Escape Room #2, https://www.learnmeded.org/mod/scorm/view.php?id=624

**Figure:**
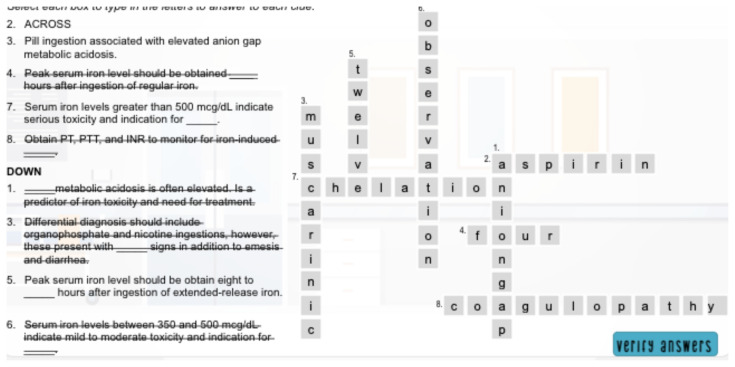
Children’s National E-Learning Team. Clue 5. In: LearnMedEd Escape Room #2, https://www.learnmeded.org/mod/scorm/view.php?id=624

**Figure:**
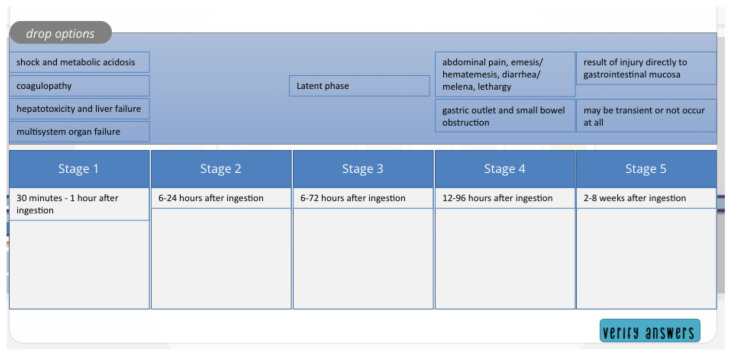
Children’s National E-Learning Team. Clue 6. In: LearnMedEd Escape Room #2, https://www.learnmeded.org/mod/scorm/view.php?id=624

**Figure:**
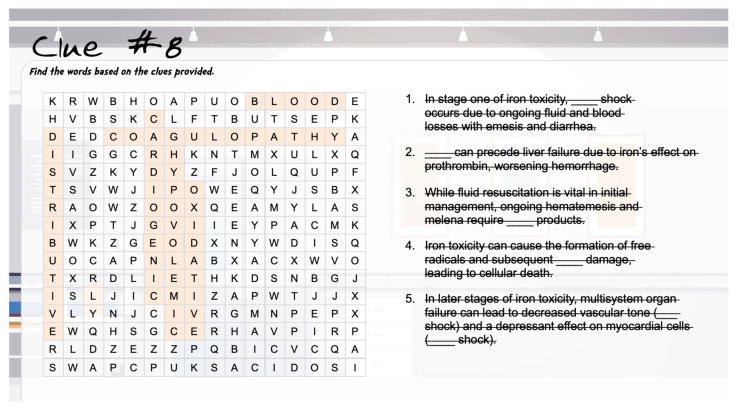
Children’s National E-Learning Team. Clue 8. In: LearnMedEd Escape Room #2, https://www.learnmeded.org/mod/scorm/view.php?id=624

**Figure:**
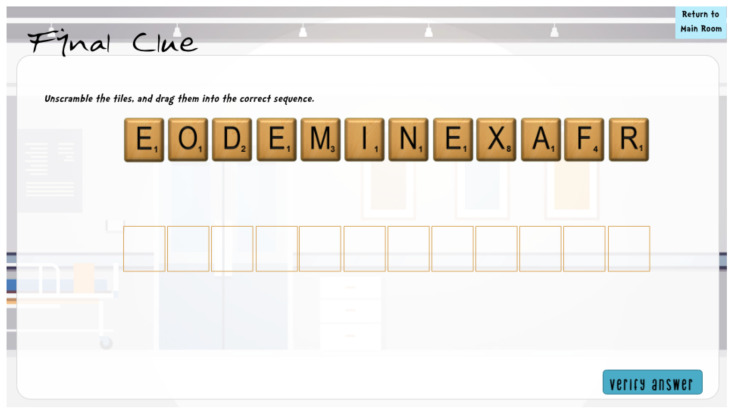
Children’s National E-Learning Team. Clue 9. In: LearnMedEd Escape Room #2, https://www.learnmeded.org/mod/scorm/view.php?id=624

**Figure:**
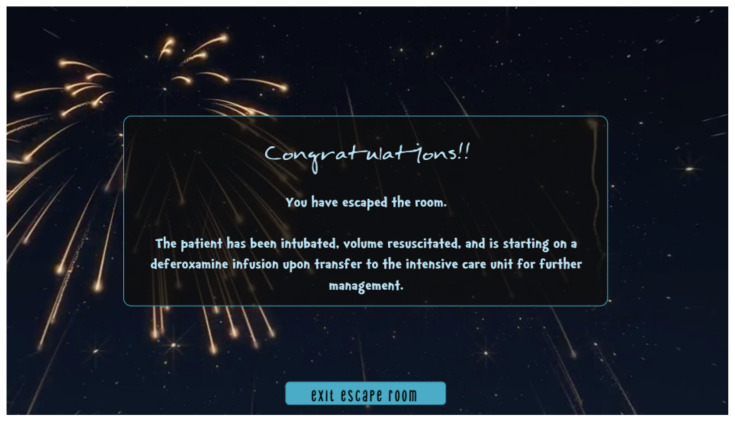
Children’s National E-Learning Team. Congratulations. In: LearnMedEd Escape Room #2, https://www.learnmeded.org/mod/scorm/view.php?id=624

## SMALL GROUPS LEARNING MATERIALS

### Appendix A Facilitator Guide

#### CASE NARRATIVE

##### Presentation

A 4-year-old female presents to the emergency department with her mom with a chief complaint of vomiting. The escape room will be opened once the initial presentation is provided, and the 60-minute timer will start. Participants will learn that the patient lives with a single mom who recently gave birth and is taking prenatal vitamins. Following primary and secondary survey assessment, an ingestion of iron pills is suspected. The participants will progress through the escape room to evaluate their knowledge of the pathophysiology and management of iron toxicity. As management and antidotes are discussed throughout the clues, the patient will have further decompensation. The final clue will be presented to “escape the room,” unlock the antidote, and ultimately save the patient.

##### Physical Exam

Vital signs: HR: 135 BPM BP: 102/62 mmHg RR: 32 BPM Oxygen: 99% Weight: 17 kg
General: active hematemesis, ill-appearing
HEENT: atraumatic, normocephalic, pupils 4 mm, equal, round, reactive to light, normal conjunctiva, extraocular movements intact, mucous membranes are moist
CV: tachycardic with no murmur or gallop
Resp: Lungs clear to auscultation; no wheezing, rhonchi, or rales
GI: Soft, tenderness to palpation in the epigastric region, normal bowel sounds, no organomegaly
Skin: Warm, dry, no diaphoresis, capillary refill 4 seconds
Neuro: awake, alert, crying; no focal deficits

##### Workup

CXR: negative
AXR: Radiopaque foreign bodies within the gastric body
EKG: sinus tachycardia

ABG: 7.31/ pCO2 32/ pO2 100
WBC: 6.1 x103/ μL
HGB: 14 g/dL
Platelets: 236 x103/ μL

PT: 11 seconds
PTT: 15 seconds
INR: 0.8

Na: 143 mEq/L
K: 3.9 mEq/L
Cl: 108 mEq/L
CO2: 15 mEq/L
BUN: 15 mg/dL
Cr: 0.7 mg/dL
Glucose: 98 mg/dL
AST: 45 U/L
ALT: 32 U/L

Tylenol: <10 Ug/mL
Salicylate: <2.5 mg/dL
Ethanol: <10 mg/DL
Urine drug screen: negative
Fe: 546 mcg/dL

#### ESCAPE ROOM

**https://www.learnmeded.org/mod/scorm/view.php?id=624**

##### Creating an account

This escape room may be accessed with an account or as a guest. In order to use as a guest, scroll down on the page and click “access as a guest.” If you would like to utilize your own unique account, you can create an account by following the instructions on the screen. Click “Escape Room #2” in order to access this escape room outlined below.

The platform will have an initial slide to orient the learner to the goals of the activity:

- The patient’s chief complaint will then be presented:
- Progression of clues: Complete clues 1+2 (in any order) → complete clues 3–5 (in any order) → complete clues 6–8 (in any order) → final clue.
  ○ If clues are opened prematurely, an error message will occur:
- A blue progress bar is at the bottom of the screen, allowing the participant to see how far they have proceeded through the escape room.
- If needed, normal lab values can be referenced in an icon in the main room at the top left corner.
- Medical chart: able to be accessed after clues 1–2, it is used to review the patient case and is not linked to any clue.
- Hint document: Will appear on the screen if learner is persistently on the same question for >2 minutes for assistance (Attached as Appendix B).
- After completing each clue, a tile will be obtained. A word scramble will pop up, and tiles will be unscrambled to unlock the final clue, as seen by example below.

##### Clue 1 (Patient)

- Review physical exam by clicking on the patient icon. General exam provided.
- May assess additional organ systems by clicking on face, chest, abdomen, and legs.
  ○ Includes audio clips, photographs, and descriptions

##### Clue 2 (Mother + newborn baby)

- Prompt: Mom asks you what is causing the patient’s current presentation. Confirming your suspicion, mom states she is currently taking the following medication.
- Participants complete cryptogram to reveal medication likely causing patient’s toxidrome.
  ○ Completed by adding 2 to prior number (ex: A=2, B=4, C=6, etc.)
  ○ Answer: Prenatal Vitamins

##### Clue 3 (X-ray)

- Prompt: “The abdominal x-ray report has resulted, noting there are 20 radiopaque pills in the gastric body. Complete the multiple-choice quiz to discuss presentation and initial management of the ingested material.”
- Question 1: Which of the following statements regarding patient presentation is true?
  ○ The patient may be tachypneic due to bronchospasm causing respiratory distress.
  ○ **The patient may be tachypneic due to metabolic acidosis, with clear breath sounds**.
  ○ The patient may present with hematemesis but will not present with melena.
  ○ The patient will not present with shock until late in the disease course.
  ○ The patient will not have signs of poor perfusion or gastrointestinal symptoms during the latent phase.
- Question 2: Which of the following is not a risk factor in this case for an accidental iron ingestion?
  ○ Unknown amount of time since ingestion.
  ○ A newborn baby in the home.
  ○ **Prenatal vitamins, since they have lower elemental iron concentrations than other iron supplements**.
  ○ The age of the patient.
- Question 3: You know you must act fast to address the toxicity caused by the ingested material; what is the best next step?
  ○ Give the patient syrup of ipecac to induce vomiting.
  ○ Give the patient activated charcoal because it has a high binding affinity to iron.
  ○ Initiate gastric lavage with deferoxamine because of the small volume needed to achieve effect.
  ○ Initiate gastric lavage with bicarbonate solution because of the small volume needed to achieve effect.
  ○ **Initiate whole-bowel irrigation because of the large number of pills noted on x-ray and serum iron level**.
- Question 4: Which of the following statements are true?
  ○ Iron is toxic because it is absorbed primarily in the stomach directly following ingestion.
  ○ If no symptoms develop within two hours of the ingestion, it is safe to discharge the patient because it is unlikely they will develop after that time.
  ○ **There is no substantial benefit in gastrointestinal decontamination in a severe iron overdose if there are no radiopaque materials on x-ray**.
  ○ Gastric lavage does not carry risk of aspiration or esophageal perforation because the patient is already experiencing emesis.
- Question 5: Gastrointestinal decontamination with whole-bowel irrigation is advised for patients with a large number of pills on x-ray and all of the following except:
  ○ **Peak serum iron levels >300 mcg/dL**.
  ○ Physical presentation suggestive of moderate or severe toxicity.
  ○ Anion gap metabolic acidosis.
  ○ Serum iron levels that increase despite deferoxamine.
  ○ Mildly symptomatic or asymptomatic patients with estimated ingestion > 60 mg/kg of elemental iron.

##### Clue 4 (Wall medicine cabinet)

- Prompt: “The nurse notifies you that mom found her prenatal vitamin bottle in her purse, and hands it to you. The bottle states that each pill contains 325 mg of ferrous sulfate. You know the toxic dose of iron is determined by the route of exposure and the iron formulation. Continue to the next screen to calculate how many mg/kg of elemental iron has been ingested orally and how this will determine management.”
- Calculation
  ○ Answer: 76
    ▪ 325mg x 20%= 65mg elemental iron → 65mg x 20 tabs = 1300mg ingested → 1300mg/17kg = 76 mg/kg.
    ▪ 20 tabs ingested was obtained in clue 3.
    ▪ 20% obtained from chart in the hint button embedded within the question (elemental iron is 20% of ferrous sulfate).
    ▪ 17 kg will be obtained from medical chart in the main room:
      **Table:**
      Contents	Elemental Fe (%)
      Ferrous fumarate	33
      Ferrous gluconate	12
      Ferrous sulfate	20
- Matching game to review risk of various iron ingestions:
  ○ Ingestions less than 20 mg/kg of elemental iron:
    ▪ Usually asymptomatic or mild symptoms
    ▪ May be discharged with return precautions
  ○ Ingestions of 20 to 60 mg/kg of elemental iron:
    ▪ Indicate moderate toxicity
    ▪ Requires observation and measurement of serum iron level to determine next steps
  ○ Ingestions ≥60 mg/kg of elemental iron:
    ▪ Associated with serious toxicity and death
    ▪ Initiate supportive management and chelation

##### Clue 5 (EKG)

- Crossword
- When a correct answer is selected, it will be crossed out as an option:
  ○ Anion gap metabolic acidosis is often elevated. Is a predictor of iron toxicity and need for treatment
  ○ Pill ingestion toxicity associated with elevated anion gap metabolic acidosis (Aspirin)
  ○ Differential diagnosis should include organophosphate and nicotine ingestions; however, these present with muscarinic signs in addition to emesis and diarrhea
  ○ Peak serum iron level should be obtained four hours after ingestion of regular iron
  ○ Peak serum iron level should be obtained eight to twelve hours after ingestion of extended-release iron
  ○ Serum iron levels between 350 and 500 mcg/dL indicate mild to moderate toxicity and indication for observation
  ○ Serum iron levels greater than 500 mcg/dL indicate serious toxicity and indication for chelation
  ○ Obtain PT, PTT, and INR to monitor for iron-induced coagulopathy

##### Clue 6 (Patient monitor)

- Prompt: Your attending asks you to describe the stages of iron toxicity to anticipate next stages in management. Drag and drop the clinical presentation with the appropriate stage of toxicity:
  ○ Stage 1:
    ▪ 30 minutes - 1 hour after ingestion
    ▪ result of injury directly to gastrointestinal mucosa
    ▪ abdominal pain, emesis/hematemesis, diarrhea/melena, lethargy
  ○ Stage 2:
    ▪ Latent phase
    ▪ 6–24 hours after ingestion
    ▪ May be transient or not occur at all
  ○ Stage 3:
    ▪ 6–72 hours after ingestion
    ▪ Coagulopathy
    ▪ Shock and metabolic acidosis
    ▪ Multisystem organ failure
  ○ Stage 4:
    ▪ 12–96 hours after ingestion
    ▪ Hepatotoxicity and liver failure
  ○ Stage 5:
    ▪ 2–8 weeks after ingestion
    ▪ Gastric outlet and small bowel obstruction

##### Clue 7 (Standing cabinet)

- Prompt: After initial evaluation and work-up, your attending states this patient is at risk for severe iron toxicity. Complete the following quiz to review why this is the case and what are the initial next steps in management.
  ○ All of the following are symptoms of severe iron toxicity except:
    ▪ **Non-anion gap metabolic acidosis**
    ▪ Signs of poor perfusion, tachycardia, tachypnea in the latent phase, even if no gastrointestinal symptoms are present
    ▪ Peak serum iron level > 500 mcg/dL
    ▪ Ingestion of >60 mg/kg of elemental iron, even if patient is asymptomatic or with mild symptoms
  ○ When considering the management of asymptomatic patients, if the serum iron level > 500mcg/dL or they have an elevated anion gap metabolic acidosis, all the next steps should be performed except:
    ▪ Ask the nurse to start a deferoxamine infusion
    ▪ Obtain a CBC, type and screen as well as PT, PTT, and INR as this patient is at risk for coagulopathy
    ▪ Obtain an AST, ALT and bilirubin level, as this patient is at risk for liver failure
    ▪ **Obtain an amylase and lipase level, as this patient is at risk for pancreatitis**
  ○ Chelation with deferoxamine is recommended for patients with any of the following signs except:
    ▪ Capillary refill of 5 seconds with persistent emesis and diarrhea
    ▪ Worsening mental status examination who is now unresponsive
    ▪ **Metabolic acidosis with an anion gap of 10**
    ▪ 4-hour serum iron level of 510 mcg/dL
    ▪ Accidental ingestion of 65 mg/kg of elemental iron
  ○ Which of the following management statements should not be followed regarding deferoxamine?
    ▪ The infusion should be started at 15m/kg/hour, increased by 5 to 10 mg/kg/hr based on the patient’s clinical course
    ▪ The infusion should be increased if there is persistent or increasing metabolic acidosis
    ▪ The infusion may be discontinued when there is resolution of symptoms such as shock, organ failure and metabolic acidosis
    ▪ **The infusion may be discontinued when the urine loses its “vin rose” color, indicating there is no longer active chelation occurring**

##### Clue 8 (Resuscitation cart)

- Prompt: The nurse notifies you that the patient’s blood pressure is dropping, the extremities are cool, and she notes melena beneath the patient who is having active hematemesis. Complete the word search to review signs and treatment of shock in this setting.
- When a correct answer is selected, it will be crossed out as an option.
  ○ In stage one of iron toxicity, hypovolemic shock occurs due to ongoing fluid and blood losses with emesis and diarrhea
  ○ coagulopathy can precede liver failure due to iron’s effect on prothrombin, worsening hemorrhage
  ○ While fluid resuscitation is vital in initial management, ongoing hematemesis and melena require blood products
  ○ Iron toxicity can cause the formation of free radicals and subsequent oxidative damage, leading to cellular death
  ○ In later stages of iron toxicity, multisystem organ failure can lead to decreased vascular tone (distributive shock) and to a depressant effect on myocardial cells (cardiogenic shock)

##### Clue 9 (Pyxis)

- Prompt: Blood products are being hung by your bedside nurse, but you want to start the patient on an antidote. Use your tiles to enter the code to unlock the medication drawer.
  ○ Tiles will be provided that were obtained after each clue, unscrambled to spell out the word: DEFEROXAMINE. The final clue will then be unlocked.
  ○ Can then click on the pyxis for the final question:
    ▪ What is the initial dose of deferoxamine (mg/hour) you are starting in this patient?
    ▪ Answer: 255
- 17kg x 15mg/kg/hr
- 17kg: Patient’s weight that can be found in the medical chart
- 15mg/kg/hr: Dosage of deferoxamine that can be found in the multiple-choice quiz in clue 7, as well as in the hint document.
- Participant will then successfully escape the room!
  ○ You have escaped the room. The patient has been intubated, volume resuscitated, and is starting on a deferoxamine infusion upon transfer to the intensive care unit for further management.

### Appendix B Key Clinical Pearls

#### Epidemiology and Etiology

- Almost 11,000 iron exposures in children less than six are reported in the US each year.^1^
- The majority of childhood iron poisoning is unintentional and results in minimal toxicity.
- More severe exposures usually involve prenatal vitamins and iron preparations that contain ferrous sulfate, which has a significantly higher concentration of elemental iron per tab than other preparations.^2^,^6^
- Tabs appeal to children because they are bright-colored and sugar-coated, resembling candy.
- Studies have shown that birth of a sibling within six months is a risk factor in those under three years of age, with the most significant risk in the first month post-partum.^6^,^7^

#### Pharmacology

- The amount of elemental iron depends on the salt form of the tab (ex., ferrous gluconate, ferrous sulfate, ferrous fumarate).^1^,^8^
- Some may be in the form of enteric-coated or time-release, which is important to consider when interpreting serum iron levels because this will delay the release and rise of iron levels.^1^
- Toxic dose is determined by the route of exposure and iron formulation.^1^,^2^,^7^
  ○ Ingestions < 20 mg/kg of elemental iron are usually asymptomatic.
  ○ Ingestions of 20–60 mg/kg of elemental iron have the potential for toxicity.
  ○ Ingestions >60 mg/kg of elemental iron are at risk for serious toxicity.
- Iron is absorbed in the duodenum and then oxidized to the ferric form (Fe3+).^7^
- Ferric iron (Fe3+) is toxic to cellular processes due to free radical production.^1^,^8^
  ○ Transferrin and ferritin normally protect cells by binding free iron; however, these are quickly saturated in acute toxicity.

#### Clinical Presentation^1^,^7^,^8^

- First stage:
  ○ 30 minutes to 6 hours after ingestion
  ○ Early gastrointestinal symptoms
    ▪ Produced by local toxicity and iron-induced damage to the GI mucosa
    ▪ Abdominal pain, emesis/hematemesis, diarrhea/melena, lethargy, metabolic acidosis
    ▪ Death in this phase is usually caused by hypovolemic shock due to capillary leak and third spacing
  ○ Patients with mild-moderate toxicity usually do not progress past this phase
- Second stage:
  ○ 6–24 hours after ingestion
  ○ Latent or relatively stable
  ○ This stage may be transient or not occur at all
- Third stage:
  ○ 6–72 hours after ingestion
  ○ Characterized by cardiovascular toxicity and coagulopathy
  ○ Shock and persistent elevated anion gap metabolic acidosis
- Fourth stage
  ○ 12–96 hours after ingestion
  ○ It does not occur in all iron toxicity cases
  ○ Hepatotoxicity and necrosis
- Fifth stage
  ○ 2–8 weeks after ingestion
  ○ Bowel obstruction/gastric outlet obstruction
    ▪ Caused by where tablets aggregate and direct tissue damage followed by scar formation

#### Workup

- Gather appropriate historical information to determine the significance and level of toxicity
  ○ Type of iron ingested and how many pills to determine the severity with the mg/kg of elemental iron.^2^
  ○ If ingestion is >6 hours, the patient is unlikely to develop symptoms unless enteric-coated or time-release formulation.^8^
- Patients with toxicity require prompt fluid resuscitation with isotonic crystalloid to avoid hypovolemic shock during early phases.^1^
- Early physical exam findings suggestive of iron toxicity.
  ○ Tachycardia, prolonged capillary refill time, tachypnea secondary to metabolic acidosis with clear lung exam, epigastric abdominal tenderness, hematemesis, and/or melena.
- Baseline laboratory evaluation should be performed to confirm iron toxicity and monitor for clinical effects.^1^,^7^
  ○ Basic metabolic panel, liver function tests, coagulation studies, complete blood count, type and screen, venous or arterial gas, peak serum iron level.
  ○ Abdomen x-ray to assess for radio-opaque tablets, especially if an unknown amount of iron ingested.
- Peak serum iron level^1^,^2^
  ○ 4–6 hours after ingestion
  ○ 8–12 hours after ingestion of enteric-coated or extended-release iron
  ○ <350 mcg/dL: minimal toxicity
  ○ 350–500 mcg/dL: mild to moderate GI symptoms, rarely develop serious toxicity
  ○ >500 mcg/dL: serious toxicity
  ○ >1000 mcg/dL: significant morbidity and mortality

#### Management

- Asymptomatic patients^1^,^2^
  ○ If the patient is asymptomatic after the observation period and serum iron level is <500 mcg/dL, able to discharge
- Mild poisoning^1^,^2^
  ○ Must differentiate systemic symptoms from local GI symptoms; many patients may have symptoms as a result of GI irritation
    ▪ These symptoms will generally not be severe enough for fluid resuscitation
    ▪ Self-limited abdominal pain, emesis, or diarrhea AND serum iron level 350 – 500 mcg/dL
  ○ Observe 6 hours after ingestion of regular iron or 12 hours after extended-release iron formulation
    ▪ If asymptomatic after this time, able to discharge
    ▪ If symptoms worsen or are persistent, start deferoxamine
- Moderate to severe poisoning
  ○ Indicative of ingestion >60 mg/kg iron, peak serum iron level >500 mcg/dL, persistent symptoms of emesis, diarrhea, and/or altered mental status^1^
  ○ Maintain euvolemia with fluid resuscitation to avoid hypovolemia in the first phase of iron toxicity
  ○ Deferoxamine^1^,^9^
    ▪ Binds with ferric iron (Fe3+) in the blood and renders it water soluble to be excreted by the kidneys
      - It gives urine a classic “vin rose” color
    ▪ Recommended for:^1^
      - Severe symptoms
      - Elevated anion gap metabolic acidosis
      - Peak serum iron level >500 mcg/dL
      - Ingested dose calculated or from visible pills on radiograph >60mg/kg elemental iron
    ▪ Continuous IV infusion at 15mg/kg/hr^1^
      - Increased 5–10 mg/kg/hr every 2–4 hours based on clinical course
        ○ Persistent presence of vin rose urine, persistent/worsening metabolic acidosis, organ failure
- Gastrointestinal decontamination^1^,^2^,^7^,^8^
  ○ Iron does not bind to activated charcoal, so it requires whole-bowel irrigation to remove a large number of pills if seen on the abdominal radiograph
  ○ Can remove iron from the intestine before absorption into tissue can occur
  ○ It is not indicated if few or no visible pills present within the stomach
  ○ Potentially indicated for
    ▪ Symptoms of moderate to severe toxicity
    ▪ High anion gap metabolic acidosis
    ▪ Peak serum iron level >500 mcg/dL
    ▪ Continued rise in serum iron level despite deferoxamine
    ▪ Asymptomatic or mildly symptomatic patients with >60mg/kg elemental iron ingested based on the number of pills seen on abdominal plain films

### Appendix C Survey

#### Virtual Escape Room Trainee Evaluation

1. I identify as
  - Male
  - Female
  - Nonbinary
2. Year in training
  - PGY-1
  - PGY-2
  - PGY-3
3. I have participated in an escape room in the past
  - Yes
  - No
4. If yes, was this for recreation or education?
  **Table:**
  	Strongly disagree	Disagree	Neither agree nor disagree	Agree	Strongly agree
  The design was easy to follow					
  This was an engaging educational model					
  The addition of puzzles did not take away from the patient case					
  This educational model created a stressful environment					
  I would prefer virtual escape room over traditional didactics					
  This was a feasible model to reinforce medical knowledge					
  I was able to identify my gaps in knowledge of this case					
  This utilized critical thinking skills to solve puzzles					
  Completing the puzzles facilitated communication between team members					
  I would participate in a virtual escape room in the future					
5. What did you enjoy about this activity?
6. What would you change about this activity?
7. Reflection: How will you apply what you learned from this session in clinical practice?
